# Larvicidal Enzyme Inhibition and Repellent Activity of Red Mangrove *Rhizophora mucronata* (Lam.) Leaf Extracts and Their Biomolecules against Three Medically Challenging Arthropod Vectors

**DOI:** 10.3390/molecules25173844

**Published:** 2020-08-24

**Authors:** Sengodan Karthi, Karthic Uthirarajan, Vinothkumar Manohar, Manigandan Venkatesan, Kamaraj Chinnaperumal, Prabhakaran Vasantha-Srinivasan, Patcharin Krutmuang

**Affiliations:** 1 Department of Biochemistry, Centre for Biological Sciences, K.S. Rangasamy College of Arts and Science (Autonomous), Namakkal, Tiruchengode Tamil Nadu 637 215, India; karthientomology@gmail.com (S.K.); uk2147karthic@gmail.com (K.U.); vinothvk02@gmail.com (V.M.); 2 Department of Biomedical Engineering, College of Engineering, Michigan State University, East Lansing, MI 48824, USA; manisscience@gmail.com; 3 Chettinad Academy of Research and Education, Kelambakkam, Chennai Tamil Nadu 603 103, India; 4 Department of Biotechnology, Periyar University, Salem Tamil Nadu 636 011, India; mullaikamaraj@gmail.com; 5 Department of Biotechnology, St. Peter’s Institute of Higher Education and Research, Avadi, Chennai Tamil Nadu 600 054, India; vasanth.bmg@gmail.com; 6 Department of Entomology and Plant Pathology, Faculty of Agriculture, Chiang Mai University, Muang Chiang Mai 50200, Thailand; 7 Innovative Agriculture Research Center, Faculty of Agriculture, Chiang Mai University, Chiang Mai 50200, Thailand

**Keywords:** mangrove, larvicidal activity, enzyme inhibition, *Rhizophora mucronata*, repellent, mosquitoes

## Abstract

The larvicidal potential of crude leaf extracts of *Rhizophora mucronata*, the red mangrove, using diverse solvent extracts of the plant against the early fourth instar larvae of *Anopheles stephensi*, *Culex quinquefasciatus* and *Aedes aegypti* mosquito vectors was analyzed. The acetone extract of *R. mucronata* showed the greatest efficacy: for *Cx. quinquefasciatus* (LC_50_ = 0.13 mg/mL; LC_90_ = 2.84 mg/mL), *An. stephensi* (LC_50_ = 0.34 mg/mL; LC_90_ = 6.03 mg/mL), and *Ae. aegypti* (LC_50_ = 0.11 mg/mL; LC_90_ = 1.35 mg/mL). The acetone extract was further fractionated into four fractions and tested for its larvicidal activity. Fraction 3 showed stronger larvicidal activity against all the three mosquito larvae. Chemical characterization of the acetone extract displayed the existence of several identifiable compounds like phytol, 3,7,11,15-tetramethyl-2-hexadecen-1-ol, 1-hexyl-2-nitrocyclohexane, eicosanoic acid etc. Enzyme assay displayed that *R. mucronata* active F3-fractions exert divergent effects on all three mosquitos’ biochemical defensive mechanisms. The plant fractions displayed significant repellent activity against all the three mosquito vectors up to the maximum repellent time of 210 min. Thus, the bioactive molecules in the acetone extract of *R. murconata* leaves showed significant larvicidal and enzyme inhibitory activity and displayed novel eco-friendly tool for mosquito control.

## 1. Introduction

Mosquitoes are central to the spreading of many infections including dengue fever, malaria, yellow fever and lymphatic filariasis, especially in areas with ecosystems that favor their breeding [1]. Mosquitoes are the primary arthropod vectors of different blood borne illness that cause millions of mortalities per year in humans [2]. Mosquitoes can also cause allergic reactions in human beings such as angioedema [3]. In India alone, there were 1.5 million cases and more than 1500 deaths caused due to malaria cases in the past decades due to mosquito vectors [4].

Malaria is chiefly spread by six mosquito species in India, with *Anopheles stephensi* the most prevalent in urban society [5]. *Aedes aegypti* are the major vectors for dengue and dengue hemorrhagic fever, which are prevalent in Africa, the Americas and India [6]. *Culex quinquefasciatus*, which often breeds in contaminated water, is the major domestic mosquito in many tropical countries and a significant lymphatic filariasis vector, with lymphatic filariasis being the highest growing vector borne illness in the tropical countries affecting more than 146 million people [7]. Larval mosquitoes are particularly striking targets for major insecticides since their breeding site is located in water, an accessible habitat [4]. Among the approaches to reduce mosquito populations and entomological inoculation rates, larvicidal represent an attractive tool to be developed for mosquito control [8,9].

Phytochemicals with higher mosquitocidal actions are now recognized as effective natural pesticides due to their exceptional larvicidal, repellent, pupicidal, and adulticidal actions [7]. Mangrove plants are generally suggested as an alternative source of bioactive ingredients, because they are thought to contain bioactive substances that are considered safe and environmentally friendly to both humans and animals [10]. The red mangrove plant, *Rhizophora mucronata* Lam. (Malpighiales: Rhizophoraceae) is indigenous to East Africa, India, Indonesia, wet tropical regions of Australia and other prominent countries of Asia [11]. The major advantage of choosing this plant is it can be collected easily and cost effective for preparing mosquitocides as compared to commercial pesticides, is easily degradable and, most importantly, has active blends of bio-active chemicals with diverse pharmacological activity. The chief tactic of the present research is to target the developmental stages, especially the Laval stage, of blood sucking pests which keen to block the adult emergence and blocks spreading dreadful diseases. Furthermore, an aim is to detect the repellent activity of the bio-active fractions of *R. mucronata* since any green based pesticides delivering higher repellent activity has higher commercial value in the market for developing better insecticides. It is crucial to investigate the toxic range of any botanical extracts or its derivatives, just as estimated for commercial pesticides, to provide safer dealings to non-targets, especially humans [3]. Thus, the present research was to investigate the chemical composition of leaf extracts of the mangrove *R. mucronata* and larvicidal, enzyme inhibitory activity and repellent against the major medically challenging pest larvae of *An. stephensi*, *Ae. aegypti*, and *Cx. quinquefasciatus*.

## 2. Results and Discussion

### 2.1. Larvicidal Activity of R. mucronata Crude Extract

Extracts isolated from the plant leaves and flowers deliver novel visions into bio-rational mosquitocidal development. Previous research displayed that botanicals are a dynamic resource with significant biological activities, such as antiviral, antifungal, phytotoxic and, most importantly, larvicidal actions [2,3,12]. An increment number of plant molecules were stated in the previous research, suggesting that there is the atmosphere possible to provide a fertile source of compounds for medicinal or therapeutic use. The synthetic chemical usage has been an essential agent for arthropod management, but they deliver harmful outcomes, including higher tenacity in the surrounding, non-target impact to other well beings, polluting the natural sources, insecticide residues being found on food, the development of insecticide resistance by the targeted pests and impact on non-targets [13]. The aquatic regions are a unique reservoir of bio-active compounds, most of them delivers inimitable structures [13]. Plant-derived chemicals are likely to be part of the future arsenal of mosquito control programs, as they can act as common toxins, reproduction and growth inhibitors or as active repellents and also oviposition deterrent [3]. Investigating the natural botanicals and their active deliverables against the mosquito larvae may ultimately prime to their practicing green based pesticides [14]. Larval management using bio-active compounds is a major vector for the effective management of blood sucking arthropods. Plants are considered to be a viable and preferred substitute to commercial larvicides for managing mosquitoes at the community level [7,14]. For these reasons, we decided to explore our local environment for possible sources of botanic larvicides that might provide effective and safe alternatives to synthetic anti-mosquito products. Here, the larvicidal action of mangrove leaf extracts was determined against three important mosquito vectors. Ethyl acetate, acetone, benzene and methanol leaf extracts of *R. mucronata* (Figure S1) were assayed against the fourth instars. Among them, acetone extracts against *Cx. quinquefasciatus* (LC_50_ = 0.129; LC_90_ = 2.8417 mg/mL), *An. stephensi* (LC_50_ = 0.378; LC_90_ = 6.035 mg/mL), and *Ae. aegypti* (LC_50_ = 0.113; LC_90_ = 1.334 mg/mL) produced the highest larval mortality (Table 1). Similarly, the lethal dosage (1.5 mg) of crude volatile oil derived from the *Piper betle* leaf exhibited a significant mortality rate against the dengue vector with more than 94% of larval mortality and the LC_50_ was observed at 0.63 mg/L [15].

### 2.2. Mortality Bioassays of Acetone Extract Fractions

The efficacy of plant extracts varies from species to species and plant parts [16]. The variability in toxic concentrations of the various plant extracts, i.e., to achieve mosquitocidal activity, may be due to differences in concentration levels between the insecticidal components of each plant; moreover, the effect of each plant extract will vary with the time of collection and season [13]. It has been reported [15] that the seed methanol extract of *Clitoria ternatea* was effective against *An. stephensi*, *Ae. aegypti*, and *Cx. quinquefasciatus* larvae with LC_50_ values of 65.2, 154.5, and 54.4 ppm, respectively. In our study, the acetone extracts of *R. mucronata* leaves were fractioned and four different fractions were collected. These four fractions were tested for larvicidal activity against three important mosquitoes in public health terms (Table 2). When testing Fraction 3, we observed the greatest larvicidal activity against *Ae. aegypti* (LC_50_ = 0.1037; LC_90_ = 1.0025 mg/mL), *An. stephensi* (LC_50_ = 0.1480; LC_90_ = 4.6480 mg/mL) and *Cx. quinquefasciatus* (LC_50_ = 0.174348; LC_90_ = 16.73929 mg/mL), respectively. Earlier studies reported that methanolic extracts of plants are larvicidal against *An. stephensi* and *Cx. quinquefasciatus* [16,17]. Kamaraj and colleagues reported the highest larval mortality in leaf petroleum ether and flower methanol extracts of *Cryptocoryne auriculata*, flower methanol extracts of *Leucas aspera*, leaf and seed methanol extracts of *Solanum torvum*, and leaf hexane extracts of *Vitex negundo* against *An. subpictus* larvae (LC_50_ = 44.21, 44.69, 53.16, 41.07, 35.32, 28.90 and 44.40 ppm; LC_90_ = 187.31, 188.29, 233.18, 142.66, 151.60, 121.05 and 192.11 ppm, respectively) and *Cx. tritaeniorhynchus* larvae (LC_50_ = 69.83, 51.29, 81.24, 71.79, 44.42, 84.47 and 65.35 ppm; LC_90_ = 335.26, 245.63, 300.45, 361.83, 185.09, 351.41 and 302.42 ppm, respectively). Ansari and colleagues examined the larvicidal activity of *Pinus longifolia* oil against three vector mosquitoes, namely, *Aedes aegypti* (LC_50_ = 82.1 ppm), *Cx. quinquefasciatus* (LC_50_ = 85.7 ppm) and *An. stephensi* (LC_50_ = 112.6 ppm).

### 2.3. Chemical Characterization of R. mucronata Extract

**Figure 1:**
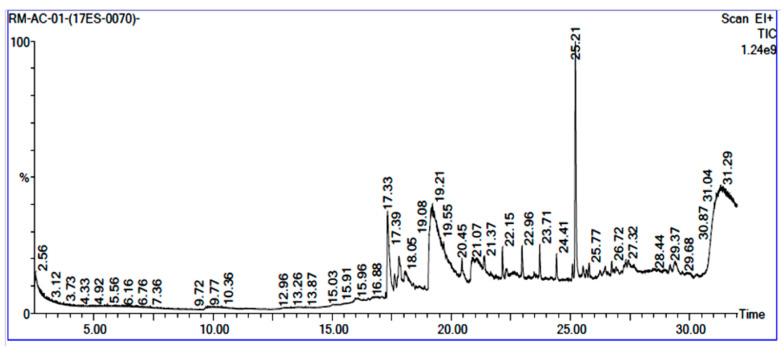
Chemical composition of GC-MS analysis using acetone leaf extract of *R. murconata*.

GC-MS analysis of acetone solvent extracts of *R. mucronata* leaves (Figure 1) showed 13 peaks representing 13 compounds. The 13 compounds were characterized and identified as shown in Table 3. phytol, 3,7,11,15-tetramethyl-2-hexadecen-1-ol, 1-hexyl-2-nitrocyclohexane, eicosanoic acid, estra-1,3,5(10)-trien-17, betaol, sulfurous acid, octadecyl 2-propyl ester, 2-heptadecenal, 1-hexyl-2-nitrocyclohexane, 17-pentatriacontene, tritetracontane, urs-12-en-28-ol and squalene. The peak area percentage was prominent in Eicosanoic Acid (38.24%) with retention time at 19.205. Since the Peak area percentage was prominent in Eicosanoic Acid and this might have played a key role in mosquitocidal activity against all the three arthropod vectors. Preliminary phytochemical screening of whole plant extracts revealed the presence of phenol, flavonoids, alkaloids, saponins, tannins, glycosides, amino acids, quinones and carbohydrates in the plant extracts (Table 4).

**Figure 2:**
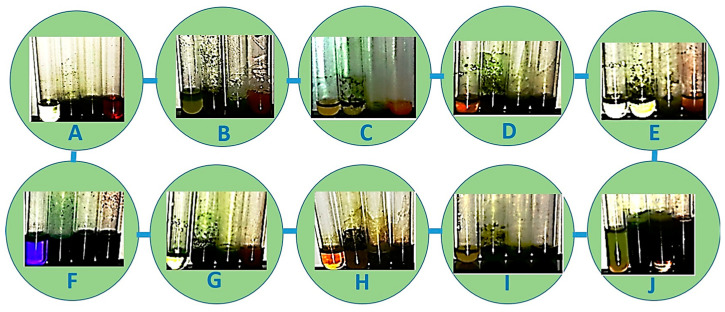
Phytochemical analysis of *R. murconata* using different solvent extracts. (**A**) Phenol; (**B**) flavonoids; (**C**) alkaloids; (**D**) saponins; (**E**) tannins; (**F**) glycosides; (**G**) protein; (**H**) amino acids; (**I**) quinones; (**J**) carbohydrates.

Plant-based compounds are known to be eco-friendly and can be used in mosquito larval control safely. Furthermore, these natural products are readily biodegradable and safe to other organisms [18]. The potent larvicidal activity of *R. murconata* could be attributed to the presence of tannins, phenols, flavonoids, saponins, glycosides and quinones (Figure 2). The isolation of these compounds, as defined here, could provide the basis for developing natural mosquitocidal products as a substitute for synthetic insecticides- or for developing mosquito repellents. The fractions obtained from the *Adhatoda vasica* were an effective larvicidal agent against the *Cx. quinquefasciatus* and *Ae. aegypti* larvae [19]. It was highly toxic to mosquito larvae and also inhibited the development of pupae. The high rate of larval mortality of mosquitoes observed at higher concentrations (250 ppm of *A. vasica*) within a 24 h exposure indicates the high toxicity of the product. The plants tested in the present study are reported to be eco-friendly and toxic to agriculturally important insect pest [20]. For example, essential oils from Citronella and Eucalyptus are often used in repellents sold under several brand names [21]. Recent studies have indicated that phytol, a precursor of synthetic vitamin E and vitamin K (which was identified in our analysis as well), exhibits antioxidant and antinociceptive effects and was cytotoxic against the MCF−7 breast cancer cell line [22].

**Figure 3:**
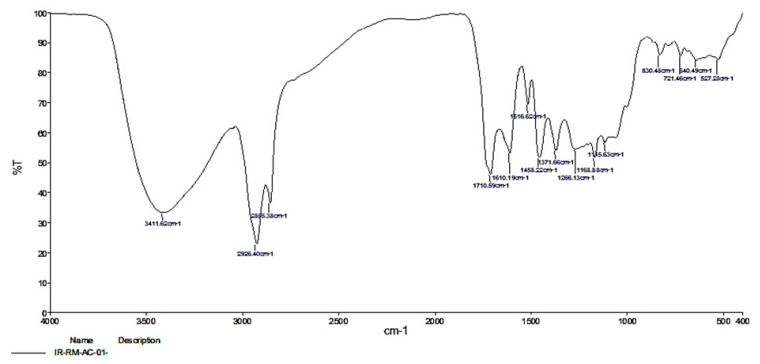
FT-IR spectrum analysis of acetone extract of *R. murconata*.

**Figure 4:**
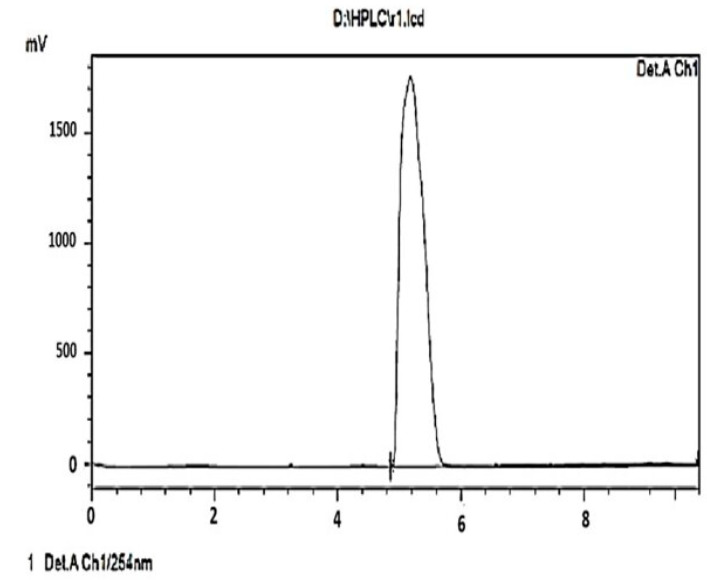
HPLC chromatogram analysis for acetone extract active F3-fractions of *R. murconata*.

The FT-IR spectrum analysis of acetone solvent extract of *R. mucronata* (Figure 3) showed 13 peaks indicating the presence of functional groups. The functional groups of the detected peak values were identified (Table 5). Alkane, Amine amd Alkyl were invariably present in acetone extracts. The column separation was eluted the different fractions of *R. mucronata*. Besides the active fraction of F3 was compared with a standard of squalene. The retention time of fraction F3 got eluted in 4.8 min at 220 nm and it was comparable to the standard peak of squalene (Figure 4). Correspondingly, Octacosane, derived from *Couroupita guianensis*, showed the highest percentage, at 31.86% peak area in the active fraction F6 and was previously shown to have toxicity to early third instars of *Cx. quinquefasciatus* (Say.) [23]. Octacosane was also shown to be present in the unsaponifiable phase of common chicory, *Cichorium intybus L.* at 1.34% peak area, exhibiting larvicidal activity against *Anopheles pharoensis* (Theobald) with LC_50_ value of 13.62 mg/kg_1 [23]. Spirostan-3,15-diol,3-(4-methylbenzenesulfo-nate) at 5.20% of peak area, was isolated by Soule et al. [24] as a spirostane glycoside from the *Solanum laxum* (Steud.) aerial parts were reported to have toxicity against the aphid, *Schizaphisgra minum* (Rondani) [25]. FractionF6 also contains hexacosane at 17.54% peak area. Hexacosane, in the leaf oil of *Solanum sarrachoides* (Sendt.), exhibits oviposition deterrence against red spidermites, (*Tetranychus evansi*) [26,27]. The sensitivity of adults of *Ae. aegypti* has been shown as resulting from the presence of 1,8-cineole, α-pinene and *p*-cymene and is correlated to the amount of 1,8-cineole in the tested extract [28]. The insecticidal activity of the essential oil of *Eucalyptus tereticornis* observed in the current work, might be explained by the presence of one of its major components (*p*-cymene) but also by a minor compound (1,8-cineole) in our extracts, which both have demonstrated insecticidal activity against *An. gambiae* [27].

### 2.4. Enzyme Assay

**Figure 5:**
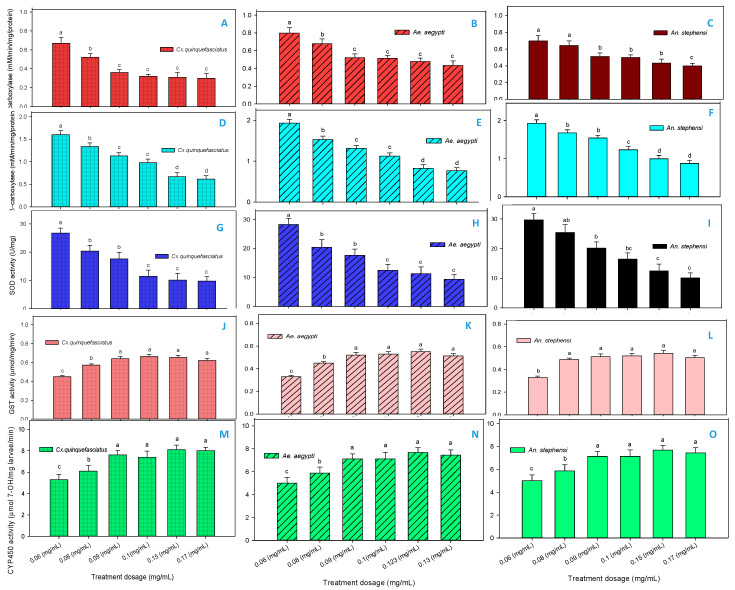
(**A**–**C**) α- carboxylestrase (**D**–**F**) β carboxylestrase (**G**–**I**) SOD activity (**J–L**) Glutathione S-transferase (**M**–**O**) CYP450 enzyme activity of *Cx. quinquefasciatus*, *Ae. aegypti*, *An. stephensi* fourth instar larvae after treatment with active acetone extract F3- fractions of *R. murconata*. Mean (± SEM) followed by the same letter in the above bars indicate no significant difference (*p* < 0.05) in a Tukey’s test.

The active acetone extract F3-fractions of *R. murconata* inhibited α-carboxylesterase activity of fourth instar larvae tested at 24 h. The activity decreased in dose dependent manner across *Cx. quinquefasciatus* (Figure 5A, F_4,20_ = 41.62; *p* < 0.0001), *Ae. aegypti* (Figure 5B, F_4,20_ = 32.12; *p* < 0.0001) and *An. stephensi* (Figure 5C, F_4,20_ = 27.22; *p* < 0.0001), respectively. Similarly, β carboxylesterase activity decreased significantly to the sub-lethal dosages of F3-fractions against all the three mosquito vectors. However, the reduction rate was prominent in *Cx. quinquefasciatus* (Figure 5D, F_4,20_ = 27.22; *p* < 0.0001) as compared to *Ae. aegypti* (Figure 5E) and *An. stephensi* (Figure 5F). Likewise, the level of SOD activity was also declined at the maximum sub-lethal dosages of all the three mosquito vectors (Figure 5G–I). However, the reduction rate was minimal at the dosage of 0.6 mg/mL in *Cx. quinquefasciatus* (F_4,20_ = 13.11; *p* < 0.0001), *Ae. aegypti* (F_4,20_ = 15.11; *p* < 0.0001) and *An. stephensi* (F_4,20_ = 17.26; *p* < 0.0001), respectively. It has been well known that many bio-insecticides affects the insect metabolism by inhibiting or stimulating the activity of digestive enzymes. Generally, SOD (superdioxide) expressed in several regions especially in anal gills of mosquitoes to catalyzes the superoxide radical dismutation into hydrogen peroxide [28].

In contrast to the above enzyme levels, GST and CYP450 enzyme regulations upregulated significantly in dose dependent manner. The enzyme ratio increased significantly initially in the lower doses (0.06 mg/mL) and relics constant at the higher sub-lethal dosages across all the mosquito vectors. Besides, the GST (Figure 5J–L) and CYP450 (Figure 5M–O) enzyme regulations uplifted significantly in *Cx. quinquefasciatus* (F_4,20_ = 11.21; *p* < 0.0001) as compared to *Ae. aegypti* (F_4,20_ = 16.71; *p* < 0.0001) and *An. stephensi* (F_4,20_ = 14.55; *p* < 0.0001). Our enzyme analysis data was interpreted to display that *R. mucronata* active F3-fractions exert divergent effects on all three mosquitos’ biochemical defensive mechanisms.

In addition to their potential for mosquito control, the *R. mucronata* extracts may exhibit enzyme inhibitory activity at currently unknown bioactivities and further studies are required to investigate the possibility of the herein identified constituents of *R. mucronata* extracts for the development of environmentally-friendly applications. Indeed, phytochemicals from plant sources may also act as insect growth regulators, repellents, ovipositor attractants, among others [26,27].

### 2.5. Repellent Activity

**Figure 6:**
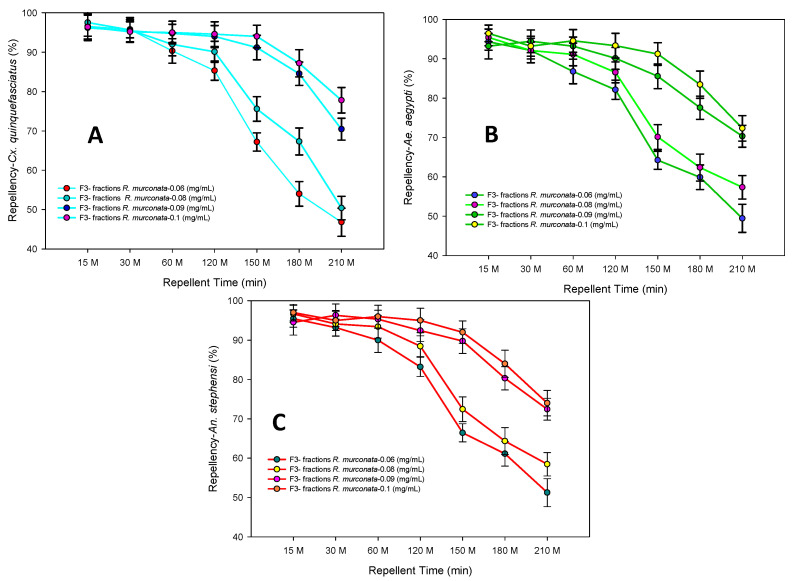
Repellency of acetone extract F3- fractions of *R. murconata* against *Cx. Quinquefasciatus* (**A**), *Ae. Aegypti* (**B**), and *An. Stephensi* (**C**). Mean (± SEM) followed by the same letter in the above bars indicate no significant difference (*p* < 0.05) in a Tukey’s test.

The active fractions of *R. mucronata* showed significant repellent activity against *Cx. quinquefasciatus* (96.4%-F_4,20_ = 24.22, *p* ≤ 0.0001), *Ae. aegypti* (94.32%-F_4,20_ = 18.82, *p* ≤ 0.0001) and *An. stephensi* (97.2%-F_4,20_ = 21.88, *p* ≤ 0.0001), respectively, at the maximum repellent dosage of 0.1 (mg/mL) at the maximum repellent time of 210 min (Figure 6. Similarly, the sub-lethal dosage (100 ppm) of crude seed extracts of *Terminalia chebula Retz.* displayed significant protection time at the maximum time of 210 min [29].

## 3. Materials and Methods

### 3.1. Plant Harvesting

The fresh leaves of *R. mucronata* were harvested from the region of Pichavaram, Rameshwaram (Latitude: 13.129991; Longitude: 79°18′46.54” E), Tamil Nadu, India, with taxonomy confirmed by an expert (Prof. Kathiresan, Department of Marine Biology, Annamalai University, Tamil Nadu) (Figure S1). The specimen voucher was preserved in the herbarium (Ref. No. BC¬/2016/Rm- 07) for further assays.

### 3.2. Crude Extract Preparation

Collected fresh leaves were air dried under shadow at room temperature for 7–10 days. The leaves (250 g) were dried and mechanically powdered using a mixer and crushed (Mixer Grinders Stylo 750) to well particle size. Leaf powdered extracts of *R. mucronata* (250 g) was prepared by using soxhlet device utilizing diverse solvents including acetone, ethyl acetate, petroleum benzene, and methanol. Further the solvents were evaporated by using rotary evaporator and the remaining were preserved under 4 °C for further assays. The total yields were observed 2.15, 1.76, 1.95 and 2.02 g, respectively.

### 3.3. Mosquitoes

*Ae. aegypti, An. stephensi*, and *Cx. quinquefasciatus* larvae were collected from the local areas of Pallipalayam (Latitude: 11.3450° N; Longitude: 77.7309° E), Tiruchengode, Tamil Nadu, India. Furthermore, the culture were maintained in the laboratory were kept in sterile vessels filled with water, maintained at 28 ± 2 °C and 75%–80% relative humidity (RH) under a fixed photoperiod (14:10 L/D). The emerged adult mosquitoes were maintained under the same conditions as the larvae.

### 3.4. Larval Mortality Assay

Larval bioassays were executed on the fourth instars of *Cx. quinquefasciatus*, *An. stephensi* and *Ae. aegypti* with diversified dosages (0.5, 0.10, 0.15, 0.20 and 0.25 mg/mL) of *R. mucronata* leaf extracts. Minimal of 20 larvae/each concentration were utilized for all the assays, and the procedure were three times replicated. The lethal dosage (LC_50_ and LC_90_) was calculated based on Probit analysis [30]. Twenty larvae of fourth instars were presented to a 200 mL glass jar supplemented with discriminating dosage of leaf extracts along with 50 mg/L of yeast extract. A group of control was also kept alone treated with methanol. Three times were replicated along with control in each replication. Mortality percentage in the treatments was corrected whenever required using Abbott formula [31].

### 3.5. Preparation of Whole-Body Homogenates for Enzyme Assay

The control and plant extract exposed fourth instar larvae were rinsed with sterile distilled water, and the adhering water was distant by using blotting tissue paper from the surface. The larval homogenized were distinct using a handy homogenizer in Eppendorf tubes containing ice-cold sodium phosphate buffer (500 μL−20 mM, pH 7.0). Homogenates were further spin for 20 min at 8000× *g* at 4 °C) and the supernatants separated were utilized for enzyme experiments. The homogenates separated were reserved at −4 °C for further experiments.

### 3.6. Carboxyl Esterase Assays

The α- and β- carboxylesterase activity was estimated using the larval extracts in phosphate potassium buffer (0.2 M; pH = 7.1) were set to (20 μL; 84 μg protein). Further, it was assorted with 500 μL buffer (0.3 mMα- or β-naphthyl acetate in 0.1 M phosphate potassium at pH 7.2 containing 1% acetone). Enzyme activity of one unit was definite as the enzyme amount essential to generate 1 μmol of α- or β-naphthol/minute.

### 3.7. Superoxide Dismutase Activity

The Superoxide Dismutase (SOD) assays were carried out by using the Superoxide dismutase determination kit (Sigma-Aldrich, Bangalore, India). Activity of SOD per 1 unit was resolute as the required enzyme to slab the increment absorbance by 50% at 440 nm.

### 3.8. Glutathione-S-Transferase Activity

A total of 250 μL of fourth instars were homogenized in sodium phosphate solution (50 mM; pH = 7.2) and spin at 10,000× *g* at 4 °C for 20 min. The Glutathione-S-Transferase (GST) assay Kit (Sigma-Aldrich, Catalog 0410, Bangalore, India) was utilized to investigate the conjugation of the thiol group of glutathione to the 1-chloro-2,4-dinotrobenzene (CDNB) substrate. Further assays were performed based on the standard protocol prescribed in the Kit. The GST activity was stated as µmol/mg protein/min substrate conjugated.

### 3.9. Cytochrome P450 Activity

Fourth instar visible to bioactive fractions3 were rinsed in sterile water and separated. Further the larvae were kept in 40 mM sodium phosphate solution with maintained pH: 7.2 and cooled earlier separation. The abdominal segments, heads, and digestive tissues were extracted. For determining the enzyme activity, carcasses were taken. The enzymes were measured by using ethoxycoumarin-*O*-deethylase existing in the body walls and it was stated as mol 7-OH/mg larvae/min.

### 3.10. Phytochemical Analysis

Chemical testing for the presence of carbohydrates, alkaloids, saponins, phenolics, tannins, terpenes and flavonoids was evaluated in the mangrove extracts and the most active extract using the standard procedure of Harborne [32].

#### 3.10.1. Phenols: Ferric Chloride Test

Extracts were treated with 3–4 drops of ferric chloride solution. Formation of bluish black color indicates the presence of phenols.

#### 3.10.2. Flavonoids Test

Ammonia solution (5 mL) was added to a portion of the crude extract followed by addition of concentrated H_2_SO_4_. Formation of a yellow coloration in the extract indicates the presence of flavonoids. The yellow coloration disappears after some time.

#### 3.10.3. Alkaloids: Wagner Reagent

Extracts were dissolved individually in dilute Hydrochloric acid and filtered. Filtrates were treated with Wagner’s reagent (1.27 g Iodine in 2 g of Potassium Iodide). The formation of a brown/reddish precipitate indicates the presence of alkaloids.

#### 3.10.4. Saponin

Half mg of the extract was shaken with 2 mL of distilled water. If foam produced persists for ten min, it indicates the presence of saponins.

#### 3.10.5. Tannin Test: Gelatin Test

To the extract, 1% gelatin solution containing sodium chloride was added. Formation of white precipitate indicates the presence of tannins.

#### 3.10.6. Glycosides Test

Minimum quantities of the extracts were hydrolyzed with hydrochloric acid for a few minutes on a water bath and the hydrolysate was subjected to the following tests.

The extracts were treated with chloroform and evaporate it to dryness. Separately, 0.4 mL of glacial acetic acid containing a trace amount of ferric chloride was added and transferred to a small test tube added with carefully 0.5 mL of concentrated sulfuric acid by the side of the test tube; blue color appeared in the acetic acid layer, indicating the presence of glycosides.

#### 3.10.7. Ninhydrin Test

To the extract, 0.25% *w/v* Ninhydrin reagent was added and boiled for few minutes. Formation of blue or blue to violet indicates the presence of amino acid.

#### 3.10.8. Benedict Test

Two mL of crude extracts were dissolved individually in 5 mL distilled water and filtered. Filtrates were treated with Benedict’s reagent and heated gently. Orange-red precipitate indicates the presence of reducing sugars.

#### 3.10.9. Starch Test

To 5 mL of the extract, a few drops of iodine was added. The presence of starch was indicated by the formation of blue color.

#### 3.10.10. Flavonoids Test

Ammonia solution (5 mL) was added to a portion of the crude extract followed by addition of concentrated H_2_SO_4_. Formation of a yellow coloration in the extract indicates the presence of flavonoids. The yellow coloration disappears after some time.

### 3.11. Repellent Assay

The active fractions F3 derived from *R. mucronata* extracts evaluated for its repellency against all the three mosquito vectors with sub-lethal dosages (0.06, 0.08, 0.09 and 0.1 mg/mL). Further repellent assay experiments were adapted from our previous research protocol Thanigaivel et al. [29].

### 3.12. GC–MS Analysis and Compound Identification

Among the different extracts (acetone, petroleum benzene, ethyl acetate and methanol) which were tested for their larvicidal toxic, and based on our preliminary tests, the highest toxicity was observed in acetone extracts of *R*. *mucronata*. Therefore, the acetone extract was used for further experiments. The isolated *R*. *mucronata* acetone extract was dissolved in (1:1) ratio with ethyl alcohol. From this, 2 μL of crude solution was dissolved in HPLC-grade methanol and subjected to GC and MS JEOL GC (Agilent Technologies 6890N, PerkinElmer, Bangalore, India) mate equipped with secondary electron multiplier. The downstream procedure of chemical characterization was carried out by our previous methodology (Agilent Technologies 6890N, PerkinElmer, Bangalore, India) [29]. The molecular weight, molecular formula and structure of the compounds of tested materials were ascertained by interpretation on mass spectrum of GC-MS using the database of the National Institute Standard and Technology (NIST).

### 3.13. FT-IR Analysis

Dried acetone extract was used for FT-IR analysis according to the combined methods reported in [33,34]. A total of 2 mg sample were mixed with 100 mg KBr (FT-IR grade) and then compressed to prepare a translucent sample disc (3 mm diameter), which was immediately kept in the sample holder. The sample was scanned and the FT-IR spectra recorded in the absorption range of 400 and 4000 cm^−1^. FT-IR analysis was performed using a Perkin-Elmer spectrophotometer (Perkin-Elmer FT-IR, Spectrum 2 Singapore, L160000A) to detect characteristic peaks, types of chemical bonds, and probable functional groups present in the sample. FT-IR peak values were recorded and the analysis was repeated twice for spectrum confirmation.

### 3.14. High Performance Liquid Chromatography (HPLC) Analysis

The HPLC (Flexar HPL, Perkin Elmer, Chennai, India) analysis was carried out with a C18 column (220 nm) (250 × 4.6 mm). Acetonitrile and water were used as mobile phase at flow rate of 1 mL/min. Peaks obtained were detected at 220 nm. Fraction F3 was analyzed and compared with a purchased standard of squalene (Sigma-Aldrich, Analytical Standard). The yield of the collected fraction dry weight is 0.57 mg.

### 3.15. Statistical Analysis

Average larval mortality data were analyzed by probit analysis to calculate the LC_50_, LC_90_, and other statistics with 95% confidence intervals of upper confidence limit (UCL) and lower confidence limit (LCL) standards and the chi-squared test. SPSS 14.0 (IBM Inc., Chicago, IL, USA) was used to analyze the data.

## 4. Conclusions

As an endnote, the phyto-chemical characterization of acetone extracts of *R. mucronata* through GC-MS analysis displayed 13 major phyto-chemicals and the individual bio-active fractions were purified and characterized through HPLC and FT-IR assays which signified that active fraction-F3 was prominent as compared to other fractions and the larvicidal activity of active fraction F3 delivers that they are lethal against larvae of all three mosquito vectors. Moreover, the enzyme assays signify that the acetone extract F3-fractions significantly drifted the enzyme pattern in all five major digestive and detoxifying enzymes. Furthermore, the sub-lethal dosage of active fraction F3 also showed significant repellent activity in a dose dependent manner across all the three arthropod vectors. From this research, it is suggested that the mangrove leaf extract of *R. murconata* contains several bioactive molecules that might be useful as eco-friendly larvicides and repellent for managing blood sucking pests of medical importance. Further functional and mechanistic studies on how these compounds exert larvicidal activity may pave the way for environmentally safe botanical insecticides to control mosquito populations.

## Figures and Tables

**Table 1:** Larvicidal activity of mangrove plant extracts of *Rhizosphora murconata* against *Ae. aegypti*, *An. stephensi* and *Cx. quinquefasciatus*. LC_50_ Lethal concentration 50% mortality, LC_90_ Lethal concentration 90% mortality, LCL: lower confidence limits, UCL: upper confidence limits, χ_2_: chi square, df: degrees of freedom.

*Species*	Solvents	LC_50_ mg/mL (95% Confidence Limit)	LC_90_ mg/mL (95% Confidence Limit)	χ^2^	df	*p*-Value
***Aedes aegypti***	Acetone	0.113	1.334	1.274	3	0.763
		(0.1935–1.147)	(0.886–2.9626)			
	Ethyl Acetate	0.305	1.037	3.078	3	0.486
		(0.125–0.556)	(1.413–8.009)			
	Methanol	0.154	1.1453	4.524	3	0.527
		(0.210–0.307)	(1.258–11.809)			
	Petroleum benzene	0.502	1.3725	5.164	3	0.498
		(0.271–1.026)	(1.803–8.368)			
***Anopheles stephensi***	Acetone	0.378	6.035	3.500	3	0.003
		(0.119–0.481)	(1.1045–11.930)			
	Ethyl Acetate	0.427	4.418	2.410	3	0.395
		(0.389–2.358)	(2.902–5.972)			
	Methanol	0.415	2.088	4.319	3	0.375
		(0.269–1.243)	(1.202–7.169)			
	Petroleum benzene	0.504	5.8592	2.311	3	0.269
		(0.304–0.895)	(1.2245–3.2839)			
***Culex quinquefasciatus***	Acetone	0.129	2.8417	1.346	3	0.865
		(0.030–0.239)	(2.700–6.302)			
	Ethyl Acetate	0.378	1.7374	2.746	3	0.468
		(0.165–0.751)	(1.861–5.499)			
	Methanol	0.295	1.0615	3.092	3	0.037
		(0.116–0.539)	(1.4216–9.4711)			
	Petroleum benzene	0.584	1.6477	2.275	3	0.284
		(0.324–1.302)	(2.0227–13.219)			

**Table 2:** LC_50_, LC_90_, and chi square analysis of larvicidal activity of *Rhizosphora murconata* acetone extract column fraction against *Ae. aegypti*, *An. stephensi* and *Cx. quinquefasciatus*. LC_50_ Lethal concentration 50% mortality, LC_90_ Lethal concentration 90% mortality, LCL: lower confidence limits, UCL: upper confidence limits, χ^2^: chi square, df: degrees of freedom. Significance at *p* < 0.05. * denotes the predominant lethal concentration dosage of Fraction F3.

Species	Column Fraction	LC_50_ mg/mL (95% Confidence Limit)	LC_90_ mg/mL (95% Confidence Limit)	χ^2^	df	*p*-Value
***Culex quinquefasciatus***	F1	0.245333	2.322	1.77430	3	0.187
		(0.159992–2.886752)	(5.002817–7.2119)			
	F2	0.341783	12.58869	3.647	3	0.476
		(0.212881–0.493197)	(4.985508–86.38909)			
	F3	0.174348 *	16.73929	3.919	3	0.528
		(0.061645–0.291515)	(5.101697–366.6364)			
	F4	0.217996	8.073846	1.82923	3	0.098
		(0.115948–0.323425)	(3.511051–45.48615)			
***Aedes aegypti***	F1	0.314289	39.94815	3.41649	3	0.461
		(0.148417–0.511897)	(9.04682–2474.55)			
	F2	0.130	1.1158	65.1	3	0.782
		(0.119–0.1561)	(0.9422–2.2120)			
	F3	0.1037 *	1.0025	4.84	3	0.521
		(0.069–0.112)	(0.8871–2.1147)			
	F4	0.20831	22.581621	3.082	3	0.391
		(0.48172–1.19820)	(20.1682–24.8216)			
***Anopheles stephensi***	F1	0.266881967	3.1525	1.938	3	0.207
		(0.0021–0.469229)	(2.9900–3.1211)			
	F2	0.31175	3.3851	0.992	3	0.034
		(0.01451–0.22655)	(2.44364–7.5935)			
	F3	0.1480 *	4.6480	3.147	3	0.218
		(0.2957–0.767397)	(3.2585–9.4680)			
	F4	1.358	5.8546	0.678	3	0.004
		(0.0198–3.2210)	(4.3215–7.5842)			

**Table 3:** Chemical characterization of acetone leaf extract of *R. murconata* through GC-MS analysis.

S.No.	Name of the Compounds	RI Polar Column Exp	Lit	RI Polar Column Exp	Lit	Peak Area %	Formula	Structure
**1**	Phytol	925	919	2622	2617	9.704	C_20_H_40_O	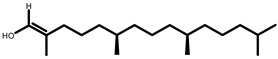
**2**	3,7,11,15-Tetramethyl-2-Hexadecen-1-ol	957	900	2114	2116	3.738	C_20_H_40_O	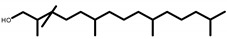
**3**	1-Hexyl-2-Nitrocyclohexane	817	814	1054	1060	1.338	C_12_H_23_O_2_N	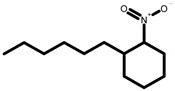
**4**	Eicosanoic Acid	913	912	2442	2445	38.246	C_20_H_40_O_2_	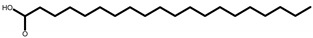
**5**	Estra-1,3,5(10)-Trien-17-Beta-ol	871	869	1145	1152	15.447	C_18_H_24_O	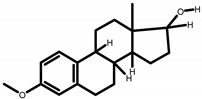
**6**	Sulfurous acid, Octadecyl 2-Propyl Ester	915	911	1231	1237	3.108	C_21_H_44_O_3_S	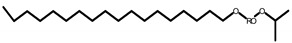
**7**	2-Heptadecenal	918	909	1174	1183	3.406	C_17_H_32_O	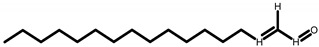
**8**	1-Hexyl-2-Nitrocyclohexane	923	916	1214	1217	5.675	C_12_H_23_O_2_N	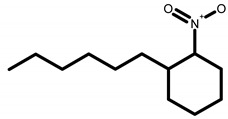
**9**	17-Pentatriacontene	929	921	1063	1066	2.450	C_35_H_70_	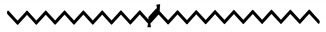
**10**	Sulfurous acid, Octadecyl 2-Propyl Ester	952	947	1118	1120	1.310	C_21_H_44_O_3_S	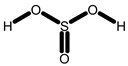
**11**	Tritetracontane	943	939	4297	4300	1.433	C_43_H_88_	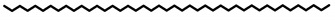
**12**	2,6,10,14,18,22-tetracosahexaene, 2,6,10,15,19,23-Hexamethyl-, (all-e)-	983	975	2814	2819	12.222	C_30_H_50_	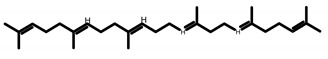
**13**	Urs-12-En-28-ol	749	748	987	992	1.923	C_30_H_50_O	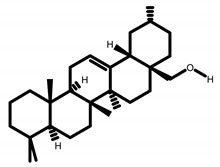

**Table 4:** Phytochemical constituent’s analysis of different solvent extracts of *R. murconata*. Where + indicates presence, - indicates absence.

S. No.	Phytochemical Test	Petroleum Benzene	Ethyl Acetate Extract	Acetone Extract	Methanol Extract
1	Phenols	+	+	+	+
2	Flavonoids	+	+	+	+
3	Alkaloids	-	+	+	+
4	Saponins	+	+	+	+
5	Tannins	+	+	+	+
6	Glycosides	+	+	+	+
7	Proteins	-	-	-	-
8	Amino Acid	-	+	+	-
9	Quinones	+	+	+	+
10	Carbohydrates	-	-	-	+

**Table 5:** FT-IR analysis of peak values of *R. mucronata* acetone extract.

S.No.	Peak (Wave Number cm^−1^)	Intensity	Bond	Functional Group Assignment
**1**	3411.62	39.09	N-H Stretch	Amine
**2**	2926.40	23.06	C-H Stretch	Alkyl
**3**	2855.38	36.55	C-H Stretch	Alkyl
**4**	1710.59	42.02	C=O Stretch	Aldehyde
**5**	1610.19	50.93	C=O Stretch	Amide
**6**	1516.62	59.60	C=C Bending	Aromatic
**7**	1458.22	49.94	C-H Bending	Alkane
**8**	1371.66	53.83	C-H Bending	Alkane
**9**	1266.13	47.76	C-N Stretch	Amine
**10**	1168.88	49.26	C-N Stretch	Amine
**11**	1115.63	53.74	C-N Stretch	Amine
**12**	830.48	80.77	C-H Bending	Aromatic
**13**	721.46	85.12	C-Cl Stretch	Alkyl Halide

## Supplementary Materials

The following are available online at https://www.mdpi.com/1420-3049/25/17/3844/s1, Figure S1: *Rhizophora mucronata* (A) whole plant and (B) stem and leaf.
