# Supplementary material for: Larvicidal Enzyme Inhibition and Repellent Activity of Red Mangrove Rhizophora mucronata (Lam.) Leaf Extracts and Their Biomolecules against Three Medically Challenging Arthropod Vectors
Source: Molecules. 2020 Aug 24;25(17):3844. doi: 10.3390/molecules25173844 (PMC7504580; doi:10.3390/molecules25173844)

Supporting information

Larvicidal Enzyme Inhibition and Repellent Activity of Red Mangrove *Rhizophora mucronata* (Lam.) Leaf Extracts and Their Biomolecules Against Three Medically Challenging Arthropod Vectors

Sengodan Karthi [1,2], Karthic Uthirarajan [1,†], Vinothkumar Manohar [1,†], Manigandan Venkatesan [3,4,†], Kamaraj Chinnaperumal [5], Prabhakaran Vasantha-Srinivasan [6] and Patcharin Krutmuang [7,8,*]

1 Department of Biochemistry, Centre for Biological Sciences, K.S. Rangasamy College of Arts and Science (Autonomous), Namakkal, Tiruchengode Tamil Nadu 637 215, India; karthientomology@gmail.com(S.K.); uk2147karthic@gmail.com(K.U.); vinothvk02@gmail.com(V.M.)
2 Division of Biopesticides and Environmental Toxicology, Sri Paramakalyani Centre for Excellence in Environmental Sciences, Manonmaniam Sundaranar University, Alwarkurichi, Tirunelveli Tamil Nadu 627 412, India
3 Department of Biomedical Engineering, College of Engineering, Michigan State University, East Lansing, MI 48824, USA; manisscience@gmail.com
4 Chettinad Academy of Research and Education, Kelambakkam, Chennai Tamil Nadu 603 103, India.
5 Department of Biotechnology, Periyar University, Salem Tamil Nadu 636 011, India; mullaikamaraj@gmail.com
6 Department of Biotechnology, St. Peter's Institute of Higher Education and Research, Avadi, Chennai Tamil Nadu 600 054, India; vasanth.bmg@gmail.com
7 Department of Entomology and Plant Pathology, Faculty of Agriculture, Chiang Mai University, Muang Chiang Mai 50200, Thailand
8 Innovative Agriculture Research Center, Faculty of Agriculture, Chiang Mai University, Chiang Mai 50200, Thailand
* Correspondence: patcharink26@gmail.com
† These authors contributed equally to this work.

Academic Editor: Giovanni Benelli
Received: 3 August 2020; Accepted: 21 August 2020; Published: date

Figure 1. *Rhizophora mucronata* (**A**) whole plant and (**B**) stem and leaf.

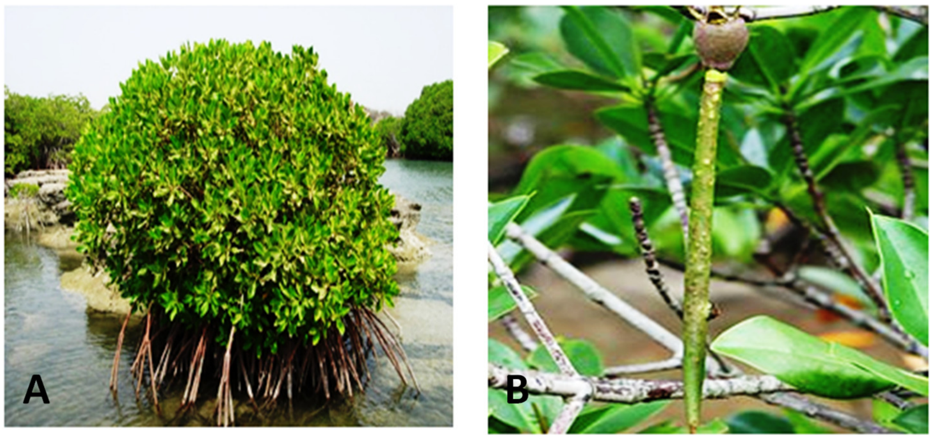

Supplement: Supplementary file 1 [file molecules-25-03844-s001.pdf]
